# Soil invertebrate diversity loss and functional changes in temperate forest soils replaced by exotic pine plantations

**DOI:** 10.1038/s41598-020-64453-y

**Published:** 2020-05-08

**Authors:** Camila Cifuentes-Croquevielle, Daniel E. Stanton, Juan J. Armesto

**Affiliations:** 1 Instituto de Ecología y Biodiversidad, Alameda 340, Santiago, Chile; 2 Universidad de Chile, Facultad de Ciencias, Santiago, Chile; 3 Department of Ecology, Evolution and Behavior, University of Minnesota-Twin Cities, Saint Paul, MN USA; 4 Cary Institute of Ecosystem Studies, Millbrook, New York, USA; 5 Departamento de Ecología, Facultad de Ciencias Biológicas, Pontificia Universidad Católica de Chile, Alameda 340, Santiago, Chile; 6 Instituto de Filosofía y Ciencias de la Complejidad, Santiago de Chile, Chile; 7 Facultad de Ciencias Naturales y Oceanográficas, Universidad de Concepción, Concepción, Chile

**Keywords:** Ecosystem ecology, Biodiversity, Forest ecology, Environmental impact

## Abstract

The global expansion of tree plantations is often claimed to have positive effects for mitigating global warming, preventing soil erosion, and reducing biodiversity loss. However, questions remain unanswered about the impacts of plantations on belowground diversity and soil properties. Here, we examine how forestry plantations of exotic trees affect critical soil functions and the composition of invertebrate assemblages, by comparing invertebrate diversity and soil physico-chemical properties between non-native *Pinus radiata* plantations, and nearby native forests in a region of extensive plantation activity in south-central Chile. We quantified differences in diversity, abundance, and community composition of soil invertebrates, as well as fundamental soil properties such as soil water content, water infiltration, nutrient status, and pH. We show that in this landscape mosaic of native forest and plantations, both soil invertebrate communities and physical soil properties differed significantly between systems, despite similar soil origins and topographies. We found a significant loss of soil carbon and a major reduction in taxonomic and functional diversity of soil invertebrates in pine plantation sites. Soil biotic and abiotic characteristics of plantations differed significantly from native forests in plantation-dominated south-central Chile, with profound consequences for ecosystem processes and resilience to future climate change.

## Introduction

Anthropogenic land cover change is considered one of the main drivers of global change in the 21^st^ century^1–3^. Since 1990, four million hectares of primary native forest have been globally lost, while at the same time the area of exotic tree plantations -which are primarily for timber and fiber production- has increased at a rate of 2% per year, accumulating 264 million hectares by 2010, and is expected to reach 300 million ha by 2020^4^. Forestry is often based on a production model that resembles agroecosystems, with costly anthropogenic subsidies and limited concerns for biodiversity loss^5^. In many countries having transitions from Mediterranean- to temperate climate (e.g., Chile, South Africa, Spain, New Zealand) vast plantations of *Pinus radiata* and several *Eucalyptus* species, have replaced the original forests^3,6^. Among the ecological consequences of the substitution of diverse native forest by massive plantations of a single exotic (non-native) timber species, is the loss of biological species richness both above and belowground, leading to a general process described as biotic homogenization^7–9^. Because of biotic homogenization, local species assemblages become depauperate, as less common and often native species tend to disappear^10^, while other species, most often non-native ones, become widespread and dominant. Biotic homogenization can also be related to the loss of specialized functional groups and the expansion of generalist, widespread species^9^, giving way to novel floristic assemblages (*sensu* Hobbs^11^).

Global analyses of changes in forest cover often assume potential ecological equivalence between tree plantations and the original native forests, in terms of carbon sequestration and forest productivity^12,13^. However, few studies have evaluated the direct impacts of large-scale replacement of native forest by exotic tree plantations on belowground invertebrate communities and on associated soil properties that, in turn, affect productivity and ecosystem functions aboveground^14^.

Evidence has shown that plant species composition determines local environmental conditions such as light incidence, temperature, soil moisture and substrate chemical quality that becomes later incorporated as detritus^15^. Furthermore, it has been reported that plant diversity is positively correlated with invertebrate diversity^16–18^. In addition, plant specific physiological traits, as litter production, defensive compounds or nutrient efficiency, have a strong effect on the rate at which soil communities perform decomposition^19,20^. Accordingly, plant species composition and soil communities are tightly related, as plants determine the quality and quantity of litter inputs available to soil communities. The loss of soil functions belowground is hard to perceive visually, but field studies have suggested strong deteriorating effects of land cover change on carbon storage, nutrient retention, and water provision^9,21,22^. All of these are significant ecosystem benefits contributing to human wellbeing^23^.

Soil invertebrates perform a wide range of functions that contribute to ecosystem health, through the maintenance of nutrient cycling, water storage and primary productivity^24^. Soil invertebrates directly or indirectly affect organic matter decomposition, the maintenance of soil structure, and can exert direct influence on plant communities through selectively feeding on roots, leaves or seeds^25^. As aboveground trophic chains are sustained by autotrophic primary production, the breakdown of belowground dead organic material performed by soil organisms, including decomposers and detritivores, provides the central input of nutrients and energy for soil processes^24^. As soils accumulate nutrients and energy through decomposition, soil communities expand and become more complex. Moreover, previous work has documented the positive interactions and feedbacks between soil invertebrates and soil microorganisms^24^ that contribute to this complexity.

Given the lack of comparative research on the soil biota and its functions in native forests and exotic forestry plantations, this work reports changes in soil invertebrate diversity and community composition between native forests and areas where the original forests were replaced by forestry plantations in El Maule region (35°S), in central Chile. This shift in land cover has been recurrent across extensive areas of central Chile beginning in the second half of the 20^th^ century^26^. In this work, we expected to identify invertebrate groups that could be useful biological indicators of soil ecological status in these two systems. To assess the functional connections between the composition of soil organisms^27^ and soil physical and chemical properties related to water and nutrient conservation, we measured these soil functions along with the assessment of the characteristics of soil biotic assemblages. Because of the importance of native and non-native forest cover, and particularly forest soils in mitigating climate change^28^, we also discuss the data on soil carbon storage in the context of the reported expansion of plantations in Chile and Mediterranean-climate regions elsewhere.

Because of its versatile requirements, fast-growing habit, and the production of medium-density softwood, suitable for a wide range of end-uses, from soil erosion control to construction and energy production^21^ *Pinus radiata* plantations have become widespread, particularly in New Zealand, Chile, Australia and Spain (Table 1). However, some environmental impacts associated with biotic homogenization of landscapes due to pine silviculture, such as the rate of spread of high-intensity fires^29,30^, soil nutrient losses, and water depletion are also well documented^31–35^. Therefore, our hypothesis is that pine plantation soils have become significantly less fertile (i.e. have lower nutrient concentrations, less water infiltration capacity, and are more acid and compacted) than native forest soils; which, in turn, is accompanied by a reduction in soil invertebrate diversity and changes in the assemblage composition, leading to a process of biotic homogenization. Based on this evidence, the notion of pine plantations as sustainable ecosystems, and the assumed equivalence of natural forests and plantations in terms of ecological processes and functions must be critically re-examined.

**Table 1:** Estimated areas *Pinus radiata* plantations around the world and the average annual change in area for the period 2008 and 2013 (data from: Mead, 2013).

Country	Estimated area (ha × 1000)	New area or loss (ha × 1000/yr)
Australia	773 (2010)	1.5
Chile	1478 (2009)	11.5
Ecuador	20 (1990)	ND
New Zealand	1545 (2011)	−11
Italy	6 (2005)	0
Spain	287 (2006)	ND
South Africa	57 (2008)	−2
Argentina	5.5 (2011)	0
Others	35	ND
Total	4207	−1

*The year of estimate is given in brackets. ND = No data for the period.

## Materials and methods

### Forests of study sites

This study was conducted in the Coastal Range of El Maule region (35°S), Cauquenes province, central Chile, which is presently covered by a novel landscape matrix dominated by pine plantations, with relatively small remnant fragments of the original native forest (less than 700 ha, all together), often restricted to steep hills and deep ravines on the Coastal Range. Forestry plantations in this region cover more than 600,000 ha, mostly represented by *Pinus radiata* trees, versus 400,000 ha of native forest, >95% of it confined to the Andes range^36^. A Mediterranean-type climate prevails over the entire study area, with rainfall concentrated during the winter months, May to August, followed by hot and dry summers^37^ extending from November to April. The Coastal Range is defined by a common basement of metamorphic rock of Palaeozoic age, with some intrusive volcanic rocks. Superimposed to this basement, younger rocks and volcanic materials of Quaternary or Tertiary age transported from the Andes, can be found^38^. The original native forests, named Maulino forest, have a canopy dominated by a mixture of deciduous and evergreen tree species, including *Nothofagus glauca* (Nothofagaceae)*, Persea lingue* (Lauraceae) and *Gevuina avellana* (Proteaceae), and less frequently *N. obliqua, Cryptocarya alba* (Lauraceae), and *Aristotelia chilensis* (Elaeocarpaceae) in some patches^39,40^. In addition, few remnant forest patches harbour narrowly distributed and locally endangered tree species, such as *N. alessandri, Pitavia punctata* (Rutaceae), and *Gomortega keule* (Gomortegaceae), all of them endemic to the Coastal Range of the study area^41^. The remaining native forests have survived a long history of human impacts, including degradation by logging and fire over the 20^th^ century, probably leading to the loss of plant species from forest fragments. Areas presently covered by pine plantations have been largely derived from fire disturbance to native forests and abandoned croplands. Nowadays, there are only two main Maulino forest remnants, both of which have been added to the Chilean National System of Protected Areas in the 1990s. These are Los Queules, with 417 ha of second-growth mixed evergreen-deciduous forest, and Los Ruiles with a total of 64 ha of predominantly deciduous forest^38^.

### Sampling sites

**Figure 1:**
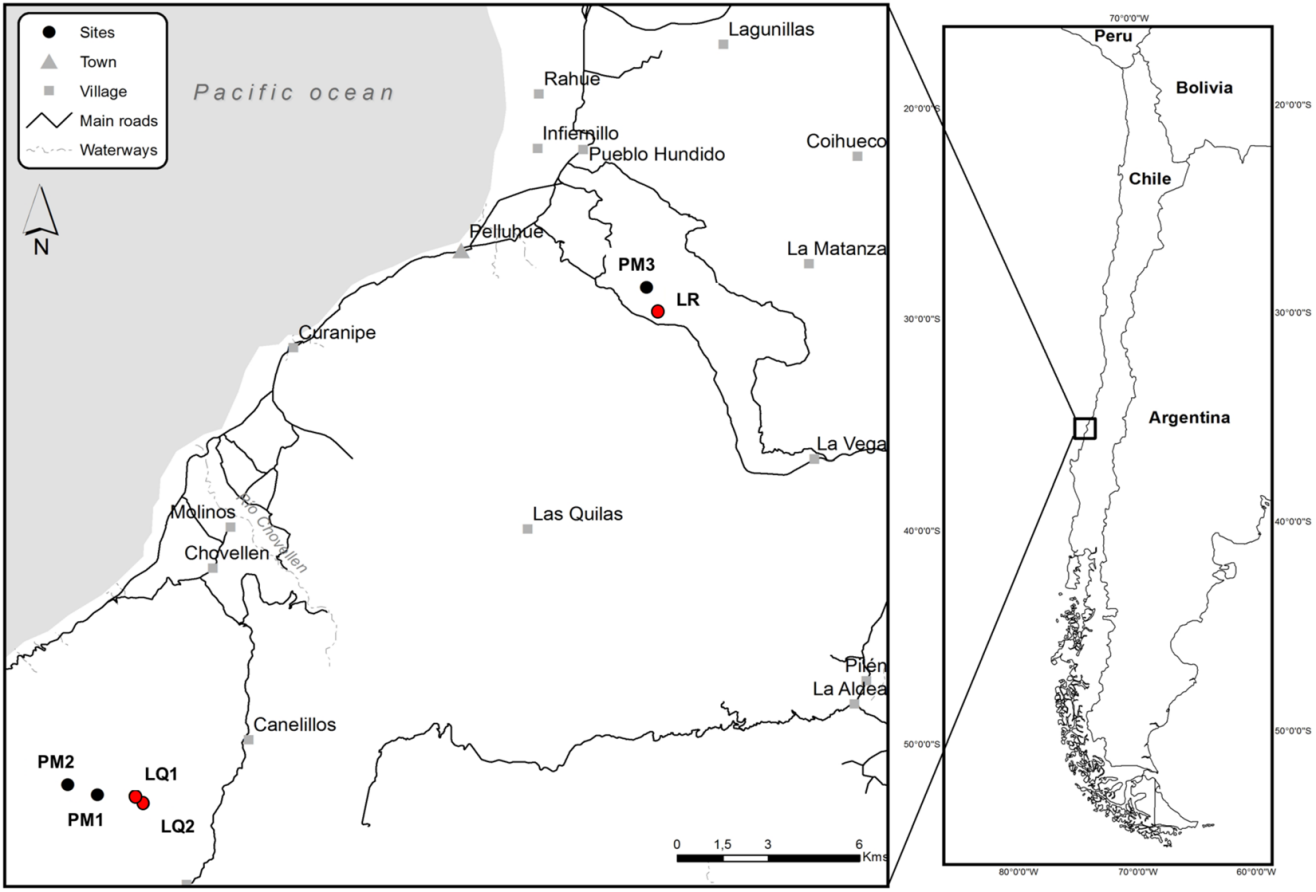
Sampling sites in Cauquenes province, El Maule Region, 35°S, in central Chile. Red dots are native forest sites (LQ1: Los Queules 1, LQ2: Los Queules 2, LR: Los Ruiles) and black dots are pine plantation sites (PM1: Pine Monoculture 1, PM2: Pine Monoculture 2, PM3: Pine Monoculture 3).

Three sites covered by secondary native forest were selected for comparison with young forestry plantations, all of them were located within two protected areas, one site was located in Los Ruiles National Reserve (35°35′57″S; 72°21′06″W) and two sites in Los Queules National Reserve (35°59′19″S; 72°41′15″W). Los Ruiles is a relatively small area of secondary and riparian forest (45 ha) dominated by winter-deciduous trees on steep hills and evergreen broad-leaved trees near the ravine. Los Queules is part of a larger fragment (600 ha) with a closed canopy of broad-leaved, evergreen forest. All three sites (Fig. 1) were selected considering a minimum distance of 50 m from any edge of native forest^42^, predominantly in areas with gentle slopes (<30°). For the comparisons, we selected three sites with similar topography, in gentle-sloped *P. radiata* plantations, less than 5 km from the nearest native forest site (Fig. 1). Sampling was also conducted at least 50 m away from patch edges. Plantations in the zone were 10–15 years old, with an average stem diameter at breast height of 26.3 ± 3.3 cm, with presence of an established understory of native and exotic shrubs derived from sprouting or post-disturbance colonization (Supplementary Fig. S1).

### Soil sampling

Three replicate plots were sampled at each of the pine plantation and native forest sites, and sampling was repeated 4 times over the course of one year to account for seasonal variation in soil invertebrate communities and properties, for a total of 72 sampling plots. New plots were established in each sampling event to avoid resampling of previously disturbed soil. Plots where distributed as follows in both native forest and pine plantation sites: We set up a 30-m long transect, following a north-south direction and at 15, 30 and 45 m from the start of the transect, we rolled two dice to determine the number of steps away from the transect where each plot would be located. We setup three 10 × 10 m plots where all canopy and understorey woody plants were identified to species, and their relative cover was estimated visually and assigned to five cover categories (0–10%, 11–20%, 21–50%, 51–75%, 76–100%)^43^. Subsequently, within each plot, we randomly selected three sampling points from where soil cores (10 cm diameter, 15 cm long) were extracted to collect the soil biota. In these same points, we estimated water infiltration by measuring the time that a defined volume of water took to infiltrate a constant soil area using the formula: Infiltration (cmh^−1^) = Q/AxT, where “Q” is the quantity of water (250 cm^3^), “A” the sampled area (78.5 cm^2^), and “T” the time (hours)^44^. From the same points, we also extracted soil cores (10 cm depth) for assessing total carbon and total nitrogen using dry combustion (Carlo-Erba Elemental Analysis). Soil pH was measured with ExStik pH Meter Extech PH100, and instantaneous soil water content was estimated by the gravimetric method.

Soil cores collected from each plot were stored at 5 °C in closed plastic bags prior to taking them to the laboratory. Soils were sieved and placed for three consecutive days in Berlese funnels for extraction of soil invertebrates^45^. After three days in the Berlese funnels, all soil remaining was manually examined under a 3× magnifying scope to collect any remaining individuals. Most groups of soil invertebrates were identified to the order level, except for better known groups, such as Coleoptera, when identifications of families was possible using available keys^46–55^.

### Data analyses

Because within-site seasonal variation was small compared to between-site variation, data from all sampling events were pooled, resulting in 12 replicate plots at each site. Sampling points within each plot are taken as pseudo-replicates for soil and invertebrate data.

The full dataset collected consisted in 216 sampling points across 72 plots, distributed in two systems: Pine plantation and native forest, with 36 plots in each (see sampling scheme in Supplementary Fig. S2). Differences in understorey plant cover and soil properties (pH, total carbon, total nitrogen, gravimetric water content, and water infiltration) among sites representing native forest and plantations, were tested using Linear Mixed Models (nlme package in R) with system as fixed effect and sites, plots and samples as nested random effects. Further, we performed permutation multivariate analysis of variance (PERMANOVA) to explore the overall difference in soil properties between pine plantation and native forest sites. Also, we performed a Principal Component Analysis to estimate which soil properties explained more variance among forest systems.

Invertebrate diversity in soil samples from pine plantations and native forest sites was estimated using Shannon’s diversity index (*H*′), and *H*′ was calculated as: *H*′ = −∑(p_i_*ln(p_i_)), where “H” is the diversity index and “p” is the proportion of species i relative to the total number of species in the sample. Comparisons between native forest and pine plantation Shannon’s diversity index were made with one-way ANOVA. Invertebrates abundance comparisons between pine plantation and native forest were made using a Generalized Linear Mixed Models with Poisson distribution.

To examine understorey plant cover and soil invertebrate diversity in all sampled sites, we used Fisher’s Alpha diversity index^56^; the relationship between soil invertebrate diversity and soil properties was tested with Linear Mixed Effects Models, with soil properties and system as fixed effects, while sites and plots as random effects.

Additionally, we computed sample-based rarefaction curves with 95% confidence intervals^57^ to compare differences in the number of invertebrate taxa (at the order level) among soil samples from native forests and pine plantation sites. Rarefaction curves compare the total number of individuals counted with repeated random sampling to the total number of taxa found in soil^58^, as it produces an accumulation curve that reaches a plateau when most of the taxa expected for the site are sampled. Accordingly, we computed the total species richness estimator Chao^57^, and to integrate both abiotic and biotic information for native forests and pine plantations, we computed a non-metric multidimensional scaling (NMDS) and the statistical analysis of similarities (ANOSIM) with Chao distance. All analyses were performed in R v.3.2.0^59^.

## Results

### Soil invertebrate communities

**Figure 2:**
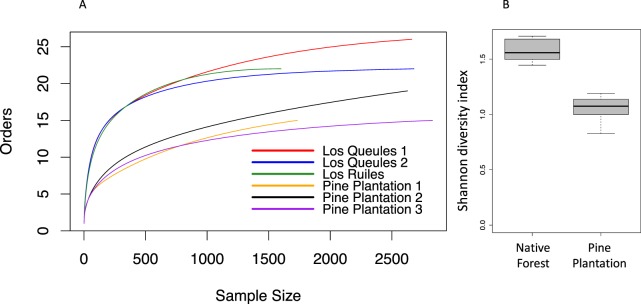
(**A**) Rarefaction analysis of the total number of invertebrate orders as a function of the total number of individuals collected from soil cores in three native forest sites and three pine plantation sites. (**B**) Shannon’s diversity index for native forest and pine plantation sites, F_1,70_ = 67.62, P < 0.001, N = 72.

Rarefaction curves showed that the accumulation of invertebrate taxa (identified at the order level) in soil samples reached different *plateau* levels in native forest and pine plantations (Fig. 2A). The asymptote indicated that sampling efforts were sufficient to characterize the local soil fauna of both systems (native forest and pine plantation). The total number of orders present, estimated by the index Chao1, was lower in soil samples from pine plantations than in soil samples from native forest sites (22 ± 2.580 and 27 ± 0.735 orders, respectively, N = 36 each). Similarly, Shannon’s diversity index based on soil invertebrate taxa was significantly lower in all pine plantation sites that in native forest sites (Fig. 2B).

We found a total of 27 orders of invertebrates in the 216 soil samples from all native forest and pine plantations sites. All the 27 orders of soil invertebrates were present in soils from native forest, while only 22 orders were found in pine plantations. Five invertebrate orders were exclusively found in native forest sites: Opilionida, Neuroptera, Trichoptera, Onycophora and Anoplura.

### Soil properties and understorey cover

Variables such as understorey plant cover, water infiltration rates and total soil carbon content differed significantly between soil samples from native forests and pine plantations (Table 2). Soils under pine plantations (N = 36) tended more acidic and considerably drier than soils under native forest cover in the same study period.

**Table 2:** Linear Mixed Models of soil properties between native forest and pine plantation sites, with random effects of sites and plots. Bold font represents significant code of p < 0.05.

Soil Property	Estimate	Standard Error	DF	t-value	p-value
Total C	−11.35	3.09	4	−3.67	**0.021**
Total N	−0.48	0.19	4	−2.51	0.065
pH	−0.11	0.05	4	−2.23	0.089
Moisture	7.86	1.08	4	−2.18	0.094
Infiltration	−16.80	4.17	4	−4.03	**0.016**
Understorey	−33.89	4.88	4	−6.94	**0.002**

Overall, understorey plant cover was significantly lower in young pine plantations than in native forests (25% versus 50%, p < 0.05, N = 72). Water infiltration and soil water content at the time of sampling were both significantly lower in pine plantation sites, with water infiltration rates ten times lower than in native forest sites. Soil water content was nearly 10% lower in pine plantation sites than in native forest sites, and soil pH was lower (therefore soil was more acid) and less variable in pine plantations, although the difference was not statistically significant. Finally, pine plantations soils had less than one half of the total carbon and total nitrogen found in soils from native forest sites, these differences were significant for C but not for N (Supplementary Fig. S3). Overall, soil properties analysed with PERMANOVA showed significant differences between native forest and pine plantations, because of differences among sites (Table 3). Principal components analysis (PCA) showed similar trends, where understorey plant cover, soil water infiltration and soil water content accounted for 92% of the variance between pine plantation and native forest sites (Supplementary Fig. S4).

**Table 3:** PERMANOVA for soil properties of native forest and pine plantation with three sites (replicates) each.

Permutation N: 9999		
Factor	F	P
System	F_1,66_ = 75.70	<**0.001**
Site	F_2,66_ = 3.77	**<0.05**
Interaction	F_2,66_ = 14.46	**<0.001**

### Biotic and abiotic controls on soil invertebrate communities

**Figure 3:**
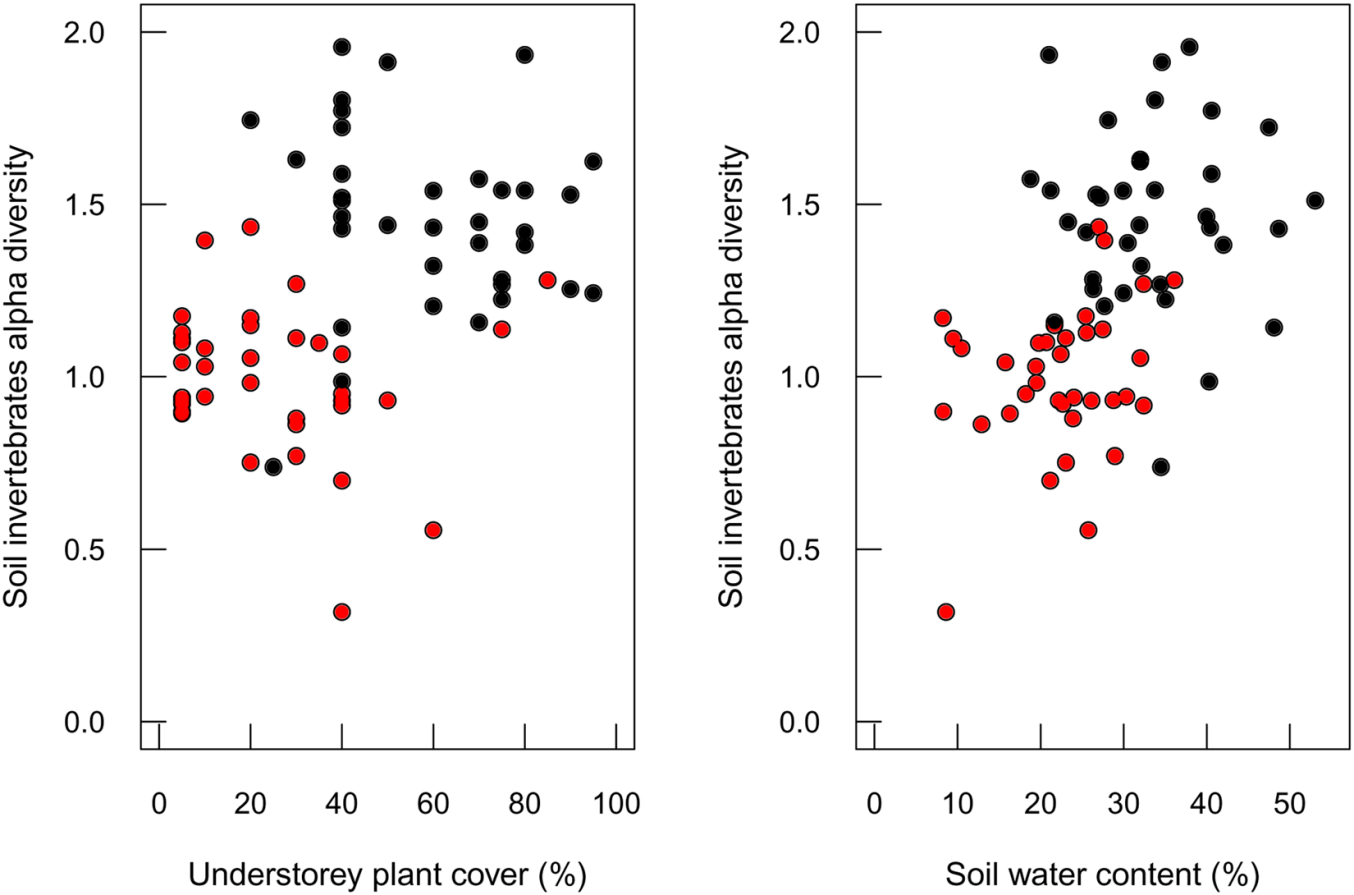
Relationship between soil invertebrates’ alpha diversity index and A) understorey plant cover (%) (SE = 0.01, DF = 64, t-value= 2.75, p < 0.001) and B) soil water content (%) (SE = 0.02, DF = 64, t-value = −2.19, p < 0.05). Black dots are native forest plots, and red dots are pine plantation plots.

Understorey plant cover in both native forest and pine plantation plots, was positively related with soil invertebrate community diversity at the plot level (Fig. 3A), documenting the strong dependence of belowground diversity on above-ground plant diversity. Moreover, the overall proportion of plant cover in the understorey was also significantly lower in plantations than in native forest plots (30% versus 50%, respectively, Supplementary Fig. S3). In addition, soil invertebrate diversity (estimated at the order level) was strongly related to soil water content on each plot (Fig. 3B). This result supports a functional relationship between soil invertebrate assemblages and soil water availability, as we show that soils of pine plantations, which have a lower soil invertebrate diversity, had significantly lower moisture content than soils under native forest cover.

### Taxonomic and functional differences between soil invertebrate communities

**Figure 4:**
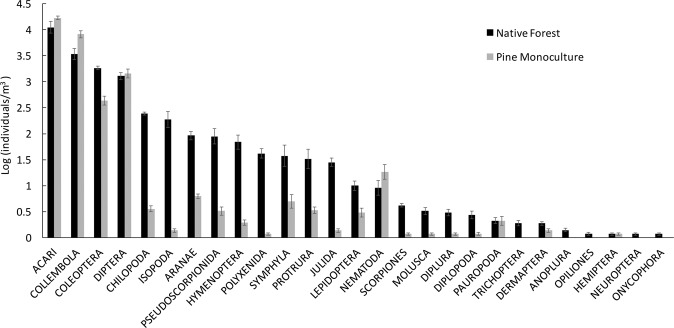
Average abundance of soil invertebrate orders found in native forest sites (black bars) and pine plantation sites (grey bars). Vertical lines indicate standard errors. Invertebrate orders are ranked according to their decreasing abundance in native forest sites. Abundances are in logarithmic scale.

**Figure 5:**
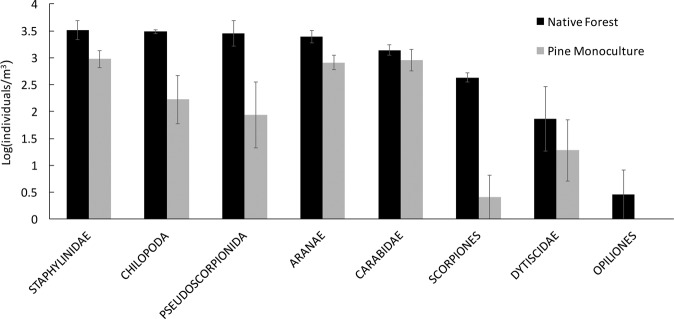
Average abundance of invertebrate predators found in soils of native forest sites (black bars) and pine plantation sites (grey bars). Vertical lines indicate standard errors. Taxa are ranked according to their decreasing abundance in native forest sites. GLM of the abundances of each group showed significant differences between pine plantation and native forest systems for Aranae (p < 0.05), Staphylinidae (p < 0.5), Chilopoda (p < 0.1) and Pseudoscorpionida (p < 0.001).

Soil community composition, in terms of invertebrate orders, differed greatly between native forest and pine plantation sites (Fig. 4). Rare invertebrate taxa in native forests, defined as taxa with less than five individuals on average per sample (i.e. Anoplura, Neuropteran, Onycophora, Trichoptera and Opilionida), were completely absent from pine plantation sites. On the other hand, widespread groups such as Collembola, Diptera and Nematoda were more abundant in soils under pine plantations than in native forest. Top predator groups (i.e. Chilopoda, Aranae, Scorpiones, Pseudoscorpionida and Staphylinidae) were significantly less abundant and had lower diversity in pine plantation than in forest sites (Fig. 5). GLM of the abundances of each group showed significant differences between pine plantation and native forest systems for all invertebrate orders but Acari (Supplementary Fig. S6).

## Discussion

Consistent differences in soil properties were observed between native forests from central Chile and pine plantation sites in the same region, over similar geological substrates and under the same climate. These differences in soils properties associated with forest cover type (native forest versus pine plantations) were in turn related to marked differences in underground invertebrate assemblages. With respect to the soil physico-chemical status and the characteristics of the soil biota, both differentiated clearly between the two ecological systems compared: native forest and pine plantations. Native forest soil was less compacted, and, despite low sample size, appears to be less acidic, and had higher water contents than soil under pine plantation. More importantly, native forest soil was consistently richer in carbon (total C) than soils from pine plantation sites. Differences among sites were evident despite the similarities in climate, substrates and past disturbance history across the landscape mosaic under study, and especially considering the short distances (<5 km) separating sampling sites under pine cover and under native forest cover. Differences in soil structure and above-ground plant communities were strongly related to contrasting diversity and composition of invertebrate assemblages. According to our analysis, lower invertebrate diversity in pine plantation sites was consistently associated with soil properties that might be promoting the loss of soil fauna (biotic homogenization). Although it is difficult to ascertain the causal relationships for the observed changes in soil invertebrate community composition and associated soil properties, the current management of pine plantations (including short rotation times and burning of the residual biomass after harvest) and the low diversity of the canopy composition (only one tree species in plantations) can drive changes in invertebrate diversity and the ecological processes that sustain it (discussed below).

### Soil properties, ecosystem processes, and above-belowground functional links

Soil properties reported here can be considered environmental proxies of soil structure and ecosystem functions that are relevant for the long-term sustainability of ecosystem productivity^15^. Water infiltration rate is an indicator of soil compaction and aeration, and water content indicates the capacity of soils to retain water in the strongly seasonal environment of central Chile, under a Mediterranean-type climate with water supply concentrated between May and August^37^. In this context, our data supports recent assessments of the indirect effects of extensive forestry plantations on water availability in similar bio-climatic locations^34,60,61^. When compared to areas dominated by native vegetation, massive forestry plantations are characterized by lower water retention, affecting its supply and quality for other downslope land uses. At the same time, the management of plantations with short rotation periods (15–25 years) are related to greater nutrient losses to stream water via sediments^34^. The impact of plantations on water storage and provision is one of the most valued and, at the same time, the most altered of all ecosystem benefits that forests in this region provide to local communities^21^.

Previous studies have reported strong soil acidification and nutrient depletion under pine plantations^22,31,33,62,63^; however, despite the rapid regional growth of forestry plantations, these effects have not been assessed in Chile before. Lower soil pH under pines has been attributed to the redistribution of mineral exchangeable cations from soil to fast-growing tree biomass; cations tend to be depleted from soil exchange sites and replaced by H^+^, thereby turning soils increasingly acid^33^. Considering that humid forest soils tend to be more acidic than other ecosystems due to increased production of carbonic acid from higher rates of autotrophic respiration^64^, the greater acidification of pine plantation soils may represent a problem for the soil biota^33^. Furthermore, soil acidification could have detrimental effects on soil fertility, also modifying soil faunal communities^65,66^ because invertebrate taxa show different pH preferences^67–69^. For example, collembolans tend to increase under greater soil acidity, which can be explained by their physiological tolerance to acid soils^65^. Accordingly, we found that, unlike most other invertebrate orders collected, collembolans and nematodes were almost twice more abundant in sites under pine plantations.

Studies of exotic plantations in other Mediterranean-climate regions have documented similar differences in entomofaunal diversity with respect to native forest remnants^70–73^. Authors have highlighted the importance of maintaining an understorey of native plant species for the conservation of invertebrate communities^74^; however, the present study shows that, in all the sites sampled, both plant species richness and cover in the understorey were significantly lower in plantations sites than in native forest plots. This difference is presumably the result of higher tree densities and stronger shading under plantations, as well as the collateral effects of harvesting operation after short rotations. Maintaining understorey cover, therefore, may require planned efforts to sustain this layer of vegetation in plantations. Such management may be at odds with the need to prevent wildfire and facilitating the harvest operation. The reduced habitat heterogeneity and lower litter diversity, characteristic of plantations, offers less shelter and lower nourishment opportunities to soil invertebrates, which could lead to a rapid biotic homogenization of soil communities. The less common groups of invertebrate taxa (“rare” taxa in Fig. 4), which were only found in native forest sites, such as Trichoptera, Anoplura, Hemiptera, Neuroptera and Onycophora, were conspicuously absent from pine plantation sites. On the other hand, the most common and widely distributed invertebrate groups (i.e., Acari, Collembola and Diptera) were more abundant in pine plantations than in native forest. The replacement of rare species by widespread ones is a known feature of the biotic homogenization process^9,10^.

Some soil invertebrates are at the top of trophic food chain, and therefore their abundances may reflect the integration of a range of underground processes^75^. In this work, top predators (i.e. Aranae, Chilopoda, Scopiones, Pseudoscorpionida and Staphilinidae) were at least twice times more abundant in native forest soils than in soils under pine plantations. Top predator abundance may be an indicator of ecosystem productivity, as energy constraints the abundance and diversity of top predators, with more productive ecosystems sustaining larger and more diverse trophic chains^76,77^. Moreover, total soil carbon content, which is often used as indicator of overall soil fertility^33^, was significantly lower in pine plantation sites, supporting the hypothesis that pine soils become less productive than native forest soils, a condition that could be a consequence of greater nutrient uptake by the fast-growing trees of *P. radiata*. Even though further analysis is needed to identify the specific linkages between the different soil invertebrate groups found in soil and their functional roles in the ecosystem, we propose here that invertebrate communities can be useful indicators of soil nutrient status because of their prominent place in the community of soil organisms and their key function in promoting soil fertility and environmental heterogeneity^5,75^.

Finally, it is important to state that even though the invertebrate community identification was very coarse (just to the level of order), a finer identification of taxa would have accentuated the differences among forest systems. Functional roles and trophic positions can be quite diverse within some orders (e.g. Acari), and the coarseness of our data fails to capture within-order differences between systems. This limitation in our study is important, but it was due to the lack of natural history information about inconspicuous groups such as soil micro-invertebrates in southern South America, and because most of the individuals sampled were at larval stages, making identification yet more difficult.

### Soil invertebrate communities as indicators of ecosystem status

Differences among sites across the forested landscape were less important for soil properties and invertebrate community composition than differences in canopy cover between native forest and pine plantation stands. This result reinforces the idea that exotic tree plantations are functionally different from floristically diverse native forests. NMDS ordination (Supplementary Fig. S4) suggests that differences in soil invertebrate communities could be explained at least partly by contrasting soil properties, such as soil nutrient contents and water infiltration rates, thus revealing a functional linkage between soil biodiversity and ecosystem processes.

If a continuum of degradation stages occurs from a “healthy forest ecosystem” to a “severely degraded forest”, the *threshold* for a regime shift from a healthy condition to a degraded condition^78^ will not depend solely on the conservation of a single remnant forest area, but also on its interactions with the surrounding landscape matrix including all other land uses. Accordingly, to ensure the maintenance of healthy ecosystems within the landscape mosaic dominated by pine plantations in central Chile, requires a better understanding of key functional connections among ecosystems, such as the continuity of underground soil processes that sustain aboveground productivity.

The present work makes a relevant contribution to resolve an important ecological question, regarding what makes a forest a forest^79^, or what makes a remnant native forest under conservation different from a managed tree plantation. This question is not only of theoretical relevance because many forestry companies and some international organizations often make no distinction between “native forest” cover and “plantation forest” cover. From the evidence discussed here, the answer to this question goes beyond the similarity in the remote perception of different cover types. We must be able to distinguish the nature of the complex system locally defined by vertical layering, above and belowground linkages, and functional biodiversity, all of which are the product of species identities, biogeochemical process, ecosystem functions, and history of land cover change^79–81^. The challenge for plantation managers is to design a production system that can be suitable for preserving both biotic diversity and ecosystem processes. In this context, we propose that soil health must be a critical aspect of the assessment of forestry systems and suggest that the composition and trophic structure of the soil biota can be a measure of such health.

### Tree plantations as carbon sinks and biodiversity reservoirs

Timber plantations are often considered as contributors to the mitigation of climate change, under the argument that the plantation of deforested areas (often termed reclamation) with fast-growing trees should enhance carbon capture. In fact, global tree cover is often quantified as a suitable indicator of the capacity of vegetation on land to act as carbon sink for climate warming mitigation^82^. Along the same line of reasoning, tree plantations may be considered as biodiversity reservoirs^70^, supporting the idea that a functional replacement of native forests by tree plantations can still provide suitable habitat for native biodiversity^12^. We argue here that there are two important caveats: first, many plantations are being placed on fertile sites that were previously forested, and secondly, native forests have been generally shown to store much more carbon, distributed in epiphytes, understory plant cover, litter and soil^21^, and this carbon remains in the ecosystem for longer periods than in tree plantations. Woody debris in native forests also provide a greater diversity of habitats for local animal communities (e.g., fallen logs and standing dead trees^83,84^). Recently, Naudts^84^ reported increased carbon emissions from young forestry plantations in Europe after 50 years of forest management, thus questioning the functional role of tree plantations as effective carbon sinks, as often argued. In this work, we show that pine plantations in central Chile are not a reservoir for underground biodiversity, originally present in native forest communities. Consequently, we propose that the massive and rapid expansion of plantations, even though canopy cover at the landscape scale may remain the same, it does not mean that one forest system is being replaced by an analogous one, because many ecological functions such as those provided underground by soil processes, yet hard to perceive by the human eye, are being significantly changed^85^.

## Conclusion

Results of the present study document that there is considerable loss of understorey plant cover, soil invertebrate diversity, and nutrients when replacing diverse native forests by single-species, exotic plantations at a massive scale. The loss of inconspicuous biodiversity from native forest ecosystems represents a significant component of the process of biotic homogenization. In this study, we show that depauperated soil communities become predominant in the short lapse that *P. radiata* plantations have been in place in the study sites (less than 40 years). Equally concerning are changes in critical ecosystem services, such as nutrient cycling, soil structure, and carbon and water storage^86^. We hope that these findings will encourage government agencies and forestry companies to critically re-examine policies that promote the rapid increase in cover of single-species plantations at the regional scale, therefore preventing serious impacts of management on belowground processes that sustain diversity and productivity.

## Supplementary information

Supplementary Information.
