# Supplementary material for: Soil invertebrate diversity loss and functional changes in temperate forest soils replaced by exotic pine plantations
Source: Sci Rep. 2020 May 8;10:7762. doi: 10.1038/s41598-020-64453-y (PMC7210289; doi:10.1038/s41598-020-64453-y)
Supplement: Supplementary file 1 — Supplementary Information. [file 41598_2020_64453_MOESM1_ESM.pdf]

# Soil invertebrate diversity loss and functional changes in temperate forest soils replaced by exotic pine plantations

Camila C. Cifuentes[1,2,6], Daniel E. Stanton[3], Juan J. Armesto[1, 2, 4, 5,7]

[1]Instituto de Ecología y Biodiversidad, Alameda 340, Santiago, Chile

[2]Universidad de Chile, Facultad de Ciencias

[3] Department of Ecology, Evolution and Behavior, University of Minnesota-Twin Cities, Saint Paul, MN USA

[4]Cary Institute of Ecosystem Studies, Millbrook, New York, USA.

[5]Departamento de Ecología, Facultad de Ciencias Biológicas, Pontificia Universidad Católica de Chile, Alameda 340, Santiago, Chile

[6]Instituto de Filosofía y Ciencias de la Complejidad, Santiago de Chile.

[7]Facultad de Ciencias Naturales y Oceanográficas, Universidad de Concepción, Chile.

Corresponding author: Camila Cifuentes C.

Phone: +56992785985

Email: camila.cifuentes@gmail.com

# SUPPLEMENTARY INFORMATION

**Supplementary Figure S1:** List of woody species collected in native forests and pine plantations sites studied.

| Species | Family | Life habit | Origin | Present in native forest | Present in pine plantations* |
|---|---|---|---|---|---|
| *Aextoxicon punctatum* | Aextoxicaceae | Tree | Native | Yes | No |
| *Amomyrtus luma* | Myrtaceae | Tree | Native | Yes | No |
| *Aristotelia chilensis* | Elaeocarpaceae | Tree | Native | Yes | Yes |
| *Azara dentata* | Flacourtiaceae | Shrub | Native | Yes | No |
| *Azara integrifolia* | Flacourtiaceae | Shrub | Native | Yes | Yes |
| *Azara petiolaris* | Flacourtiaceae | Shrub | Native | Yes | Yes |
| *Azara serrata* | Flacourtiaceae | Shrub | Native | Yes | Yes |
| *Bomarea salsilla* | Alstromeriaceae | Vine | Native | Yes | Yes |
| *Cassia stipulacea* | Leguminosae | Shrub | Native | Yes | No |
| *Chusquea* sp. | Poaceae | Shrub | Native | Yes | Yes |
| *Cissus striata* | Vitaceae | Vine | Native | Yes | Yes |
| *Citronella mucronata* | Icacinaceae | Tree | Native | Yes | No |
| *Cryptocarya alba* | Lauraceae | Tree | Native | Yes | Yes |
| *Dioscorea brachybotrya* | Dioscoreaceae | Vine | Native | Yes | Yes |
| *Dioscorea* sp. | Dioscoreaceae | Vine | Native | No | Yes |
| *Elytropus chilensis* | Apocynaceae | Vine | Native | Yes | No |
| *Escallonia pulverulenta* | Rosaceae | Shrub | Native | Yes | Yes |
| *Gaultheria insana* | Ericaceae | Shrub | Native | Yes | Yes |
| *Gevuina avellana* | Magnoliopsidae | Tree | Native | Yes | Yes |
| *Greigia sphacelata* | Bromeliaceae | Shrub | Native | Yes | Yes |
| *Lapageria rosea* | Philesiaceae | Vine | Native | Yes | Yes |
| *Lardizabala biternata* | Lardizabalaceae | Vine | Native | Yes | Yes |
| *Laureliopsis philippiana* | Lauraceae | Tree | Native | Yes | No |
| *Lithraea caustica* | Anacardiaceae | Tree | Native | Yes | Yes |
| *Lomatia dentata* | Proteaceae | Tree | Native | Yes | No |
| *Lomatia hirsuta* | Proteaceae | Tree | Native | Yes | No |
| *Luma apiculata* | Myrtaceae | Tree | Native | Yes | Yes |
| *Luma chequen* | Myrtaceae | Tree | Native | Yes | Yes |
| *Maytenus boaria* | Celastraceae | Tree | Native | No | Yes |
| *Muehlenbeckia hastulata* | Polygonaceae | Vine | Native | No | Yes |

| | | | | | |
|---|---|---|---|---|---|
| *Mutisia ilicifolia* | Asteraceae | Vine | Native | Yes | No |
| *Myrceugenia obtusa* | Myrtaceae | Tree | Native | Yes | No |
| *Myrceugenia parvifolia* | Myrtaceae | Shrub | Native | Yes | No |
| *Nothofagus alessandri* | Nothofagaceae | Tree | Native | Yes | No |
| *Nothofagus glauca* | Nothofagaceae | Tree | Native | Yes | Yes |
| *Nothfagus obliqua* | Nothofagaceae | Tree | Native | Yes | No |
| *Persea lingue* | Lauraceae | Tree | Native | Yes | No |
| *Peumus boldus* | Monimiaceae | Tree | Native | Yes | Yes |
| *Pinus radiata* | Pinaceae | Tree | Exotic | No | Yes |
| *Pitavia punctata* | Rutaceae | Tree | Native | Yes | No |
| *Podanthus mitiqui* | Asteraceae | Shrub | Native | No | Yes |
| *Podanthus ovatifolius* | Asteraceae | Shrub | Native | No | No |
| *Proustia pyrifolia* | Asteraceae | Vine | Native | No | Yes |
| *Quillaja saponaria* | Rosaceae | Tree | Native | Yes | Yes |
| *Raphitamnus spinosus* | Verbenaceae | Tree | Native | Yes | No |
| *Cytisus striatus* | Fabaceae | Shrub | Exotic | Yes | Yes |
| *Rhamnus diffusus* | Rhamnaceae | Tree | Native | Yes | No |
| *Ribes punctatum* | Grossulariaceae | Vine | Native | Yes | No |
| *Rosa eglanteria* | Rosaceae | Shrub | Native | Yes | Yes |
| *Rubus* sp. | Rosaceae | Vine | Exotic | No | Yes |
| *Senna stipulacea* | Fabaceae | Shrub | Native | Yes | No |
| *Sophora macrocarpa* | Fabaceae | Shrub | Native | Yes | Yes |
| *Teline monspessulana* | Fabaceae | Shrub | Exotic | No | Yes |
| *Ugni candollei* | Myrtaceae | Shrub | Native | Yes | Yes |

*All woody species present in pine plantations other than *P. radiata* were limited to understorey.

**Supplementary Figure S2**: Scheme of the comparative sampling design, consisting of two forest systems (Native Forest and Pine Plantation), with three sites for each system (Los Queules 1, Los Queules 2, Los Ruiles, Pine Plantation 1, Pine Plantation 2 and Pine Plantation 3). On each site, 12 plots were made, and 3 samples were taken from each plot. This accounts for a total of 216 soil samples.

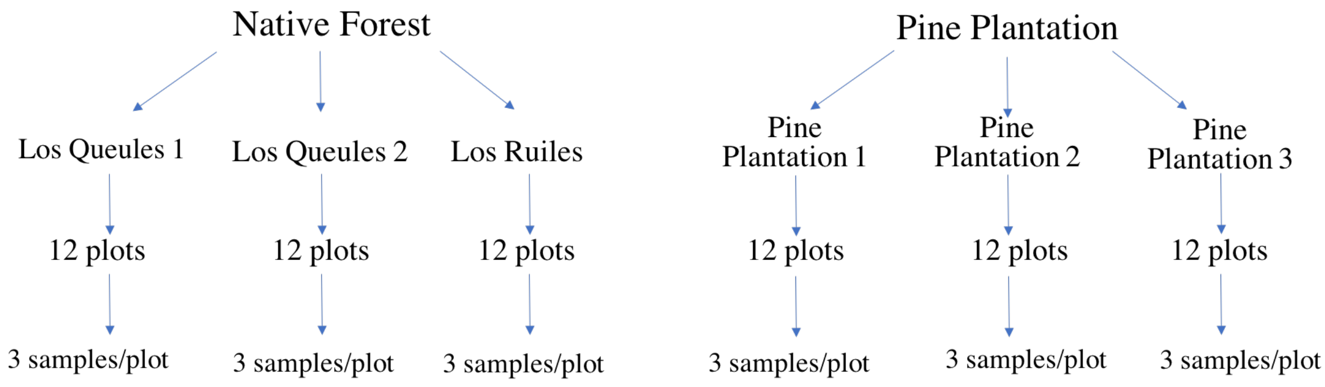

**Supplementary Figure S3:** Box plot comparison of soil properties between native forest and pine plantation sites. Grey boxes represent the distance between the 1st and the 3rd quartile, the thick black lines are the medians and the vertical dashed lines are the minimum and maximum values, in each case. A) Understory plant cover (%), $p<0.01$; B) Water infiltration rate (cm/h), $p<0.05$; C) Water content (%), $p=0.094$; D) pH, $p=0.089$; E) Total carbon, $p<0.05$; F) Total nitrogen, $p=0.065$. N=72 plots.

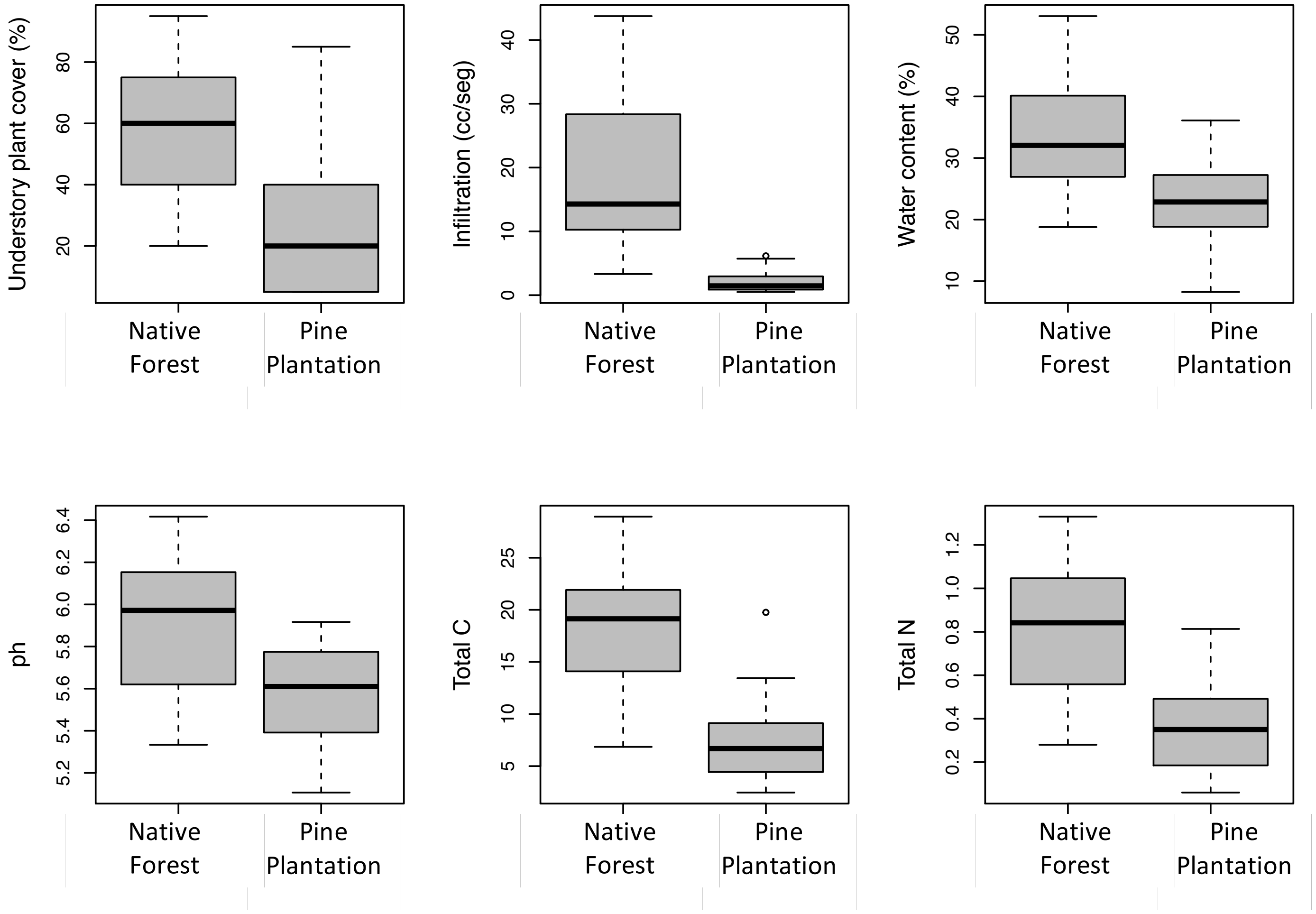

**Supplementary Figure S4:** Principal Components Analysis (PCA) of soil properties in 72 plots of pine plantation and native forest sites. Blue numbers represent the plots, and the red arrows are the soil properties. Components 1 and 2 explain 92% of the variance, while Understorey, Infiltration and Moisture had the higher loadings for the Component 1 (0.94, 0.293 and 0.129, respectively).

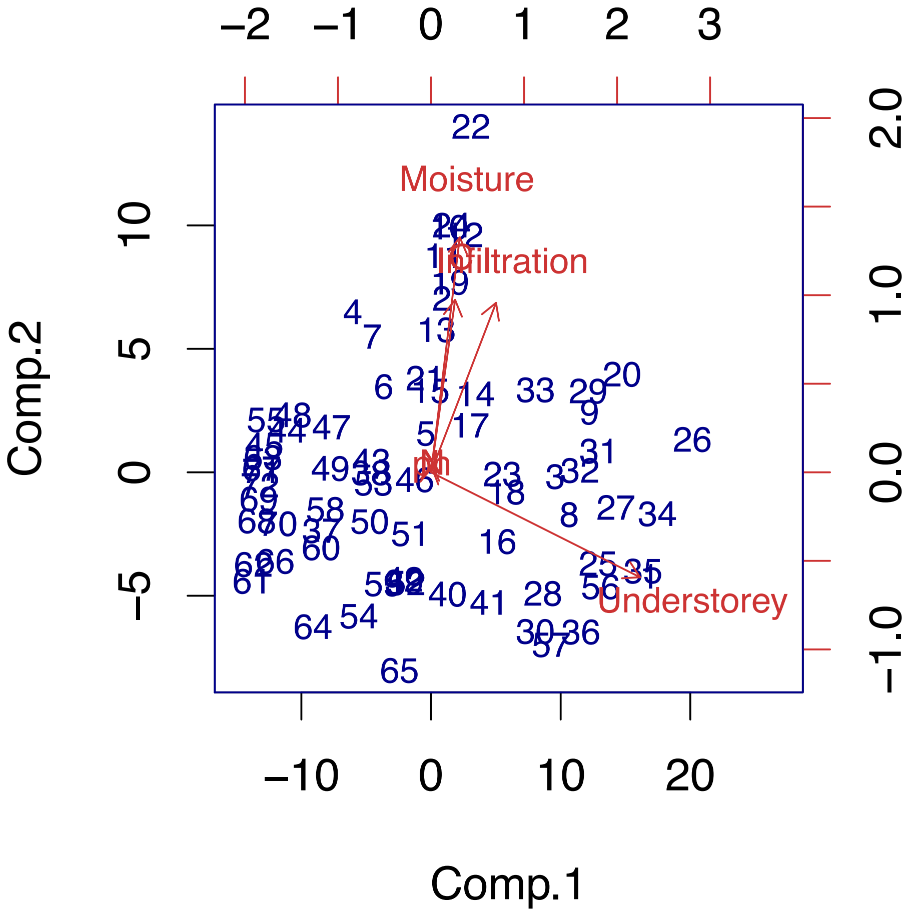

**Supplementary Figure S5:** NMDS ordination of soil invertebrate orders sampled (in red letters) and soil abiotic physical properties (blue vectors). We also calculated ANOSIM statistic to test these differences in invertebrate communities, which resulted signifficant (R=0.421, P<0.001). Furthermore, when soil properties were represented in the ordination, pH, infiltration rates, and understorey plant cover were the main factors differentiating between soil invertebrate communities. The invertebrate groups that accounted for the main differences between soil communities were Onycophora, Opilionida, Diplopoda, Scorpiones, Diptera and Nematoda.

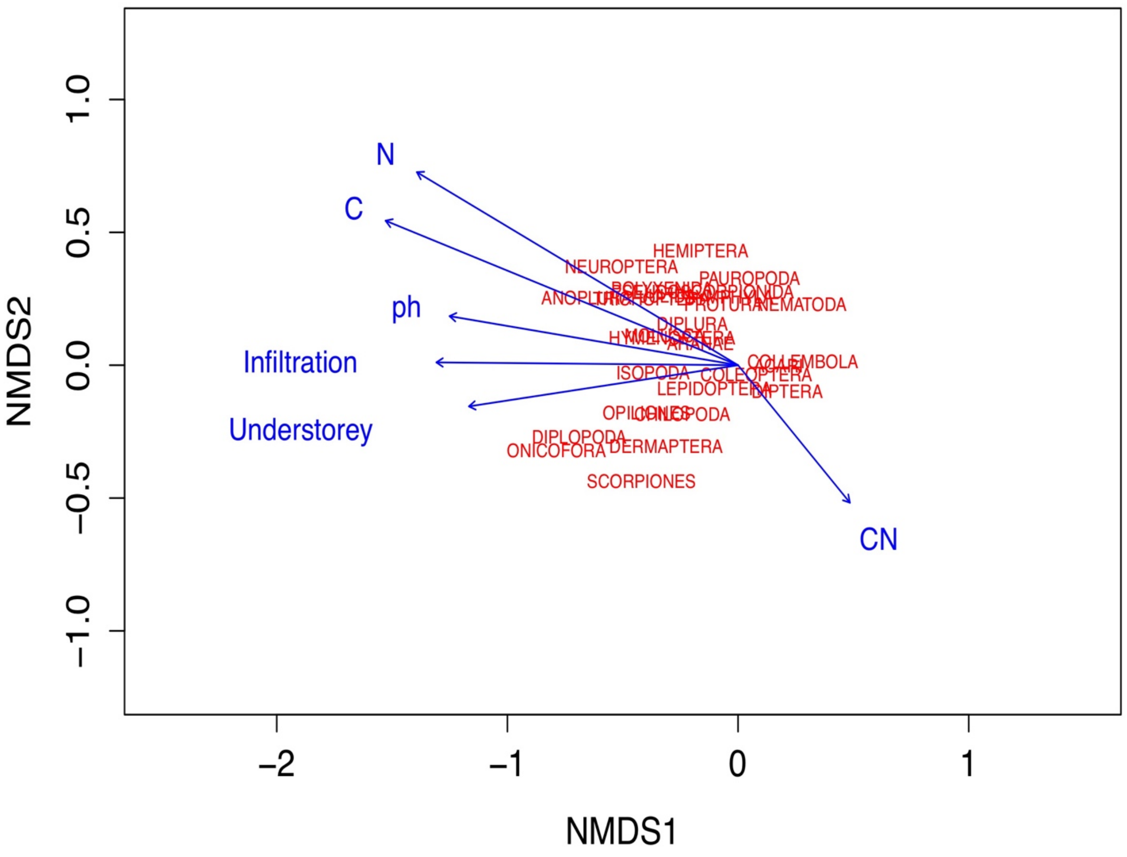

**Supplementary Figure S6:** General Lineal Model of invertebrate abundances at the order level, between pine plantation and native forest sites. Model was made with nested effects in System, Site and Plot and Poisson distribution.
Significance code: * <0.05, **<0.01, *** < 0.001, n.s= non-significant.

| | Estimate | Std. Error | z value | Pr(>\|z\|) |
|---|---|---|---|---|
| (Intercept) | 1.916982 | 0.05972 | 32.099 | *** |
| ACARI | 0.088304 | 0.077145 | 1.145 | N.S |
| ANOPLURA | -2.201098 | 0.57786 | -3.809 | *** |
| ARANAE | -2.339293 | 0.129161 | -18.111 | *** |
| CHILOPODA | -2.410817 | 0.124212 | -19.409 | *** |
| COLEOPTERA | -2.180436 | 0.060632 | -35.962 | *** |
| COLLEMBOLA | 0.241719 | 0.030909 | 7.82 | *** |
| DERMAPTERA | -2.530556 | 0.500471 | -5.056 | *** |
| DIPLOPODA | -1.963286 | 0.317447 | -6.185 | *** |
| DIPLURA | -2.524916 | 0.353999 | -7.133 | *** |
| DIPTERA | -1.571044 | 0.057032 | -27.547 | *** |
| HEMIPTERA | -2.699614 | 1.00026 | -2.699 | ** |
| HYMENOPTERA | -1.99431 | 0.103016 | -19.359 | *** |
| ISOPODA | -1.44794 | 0.086147 | -16.808 | *** |
| JULIDA | -2.065525 | 0.139715 | -14.784 | *** |
| LEPIDOPTERA | -2.20387 | 0.21387 | -10.305 | *** |
| MOLUSCA | -2.222346 | 0.289276 | -7.682 | *** |
| NEMATODA | -2.274573 | 0.193289 | -11.768 | *** |
| NEUROPTERA | -2.895272 | 1.000308 | -2.894 | ** |
| ONYCOPHORA | -2.308298 | 1.000678 | -2.307 | * |
| OPILIONES | -2.316467 | 0.707646 | -3.273 | ** |
| PAUROPODA | -1.196125 | 0.201132 | -5.947 | *** |
| POLYXENIDA | -1.766025 | 0.106786 | -16.538 | *** |
| PROTURA | -0.409568 | 0.063106 | -6.49 | *** |
| PSEUDOSCORPIONIDA | -1.806429 | 0.099986 | -18.067 | *** |
| SCORPIONES | -2.272119 | 0.316876 | -7.17 | *** |
| SYMPHYLA | -1.294876 | 0.084679 | -15.292 | *** |
| TRICHOPTERA | -2.673985 | 0.447646 | -5.973 | *** |
